# Semantic Explanation for Malaria Diagnosis: Comparing Human and Machine Generated Annotations for *Plasmodium* Species and Life-Stage Features

**DOI:** 10.1109/OJEMB.2026.3690482

**Published:** 2026-05-05

**Authors:** Kamal B. Jillahi, Zainab S. Usman, Charles Nche

**Affiliations:** Department of Computer ScienceAmerican University of Nigeria 108016 Yola 640101 Nigeria; Department of Computer Science and Software EngineeringAmerican University of Nigeria 108016 Yola 640101 Nigeria; St. Mary's University Institute Tiko 237 Cameroon

**Keywords:** Image annotation, medical diagnosis, reasoning, semantic segmentation, web ontology language

## Abstract

*Goal*: Accurate detection of malaria parasites using convolutional neural networks (CNNs) relies heavily on the quality of training annotations, yet creating quality annotations is both time-consuming and difficult to scale in high-burden, resource-limited settings. To address this challenge, we propose a method of annotating thin-smear blood images for the semantic segmentation of *Plasmodium* species, their developmental stages. Using a balanced collection of images from the Malaria Parasite Image Database, we trained identical SegNet models under three matched annotation regimes: expert manual labeling, SegNet-Only, and SegNetOntology where predictions are refined through biomedical ontological reasoning. Model performance was assessed not only for segmentation quality but also for how well each approach captured biologically meaningful information and for its interpretability as judged by clinicians. The proposed method produced results comparable to those achieved by expert annotations and clearly outperformed the baseline SegNet-only model in terms of biological consistency and clinical trustworthiness. The method successfully filtered out 5.7 of invalid AI-generated annotations by identifying semantic contradictions, ensuring the final training dataset adhered strictly to established biological constraints. Clinicians found the outputs from the proposed model nearly as reliable and understandable as those generated from expert annotations. These findings show that embedding formal biomedical knowledge into the annotation process can substantially reduce the cost and effort of creating training data while maintaining diagnostic accuracy and interpretability.

## I. Introduction

Artificial intelligence (AI) has rapidly evolved in medical diagnostics over the past decade, especially through deep learning enhancing image analysis [1], [2]. In malaria detection, automated classification of *Plasmodium* parasites in blood smears has gained traction, particularly using Convolutional Neural Networks (CNNs), which often match the accuracy of trained microscopists [3], [4]. Nonetheless, challenges regarding the interpretability and generalizability of CNN models remain, questioning their trustworthiness and potential for clinical use [5], [6].

Central to developing CNN-based diagnostic tools is the need for high-quality annotated datasets [7]. Traditional methods rely on domain experts to meticulously annotate parasite features like species type and intra-erythrocytic life stages. While this ensures biological accuracy, the process is time-intensive and limits scalability, especially in low-resource environments [9]. Additionally, inter-observer variability in human annotations can introduce subjectivity, which may affect model training [10].

Machine-generated annotations are becoming a viable scalable alternative due to advances in self-training, weak supervision, and model-assisted strategies [11]. These methods can utilize pre-trained models to create synthetic labels for new datasets, enhancing annotation efficiency and consistency [12]. However, concerns regarding the biological validity, diagnostic fidelity, and interpretative quality of these annotations exist, especially in critical areas like malaria diagnosis where detailed morphological distinctions are vital [13]. Additionally, the semantic explainability of the model's outputs, ensuring that predictions are justified in biologically relevant terms, is essential for the effective deployment of AI in clinical and public health settings [14].

This study proposes an automated annotation method to compare the effectiveness of machine-generated annotations for training CNN models in identifying *Plasmodium* species and their life stages in thin blood smear images. It employs semantic reasoning on biomedical ontologies to filter biologically implausible classification, then assess how each annotation strategy impacts predictive performance, interpretability, and clinical relevance. The research specifically investigates whether models trained on synthetic annotations from a pre-trained model can match or exceed the performance and clarity of those trained on expert-annotated data, reflecting current efforts in weak supervision and auto-generated annotation in biomedical imaging [15], [16].

The findings will contribute to the growing body of knowledge on scalable and transparent AI in global health, while also offering practical guidance on dataset preparation strategies in contexts constrained by human expertise. Ultimately, the study aspires to advance the development of interpretable, biologically grounded, and deployable AI systems that support accurate malaria diagnosis in both research and real-world clinical settings.

### A. Differentiation From State-of-the-Art Methods

Deep learning in medical imaging has demonstrated expert-level efficacy for certain tasks [5], [6], but its dependence on large, manually annotated datasets is a significant barrier [9], [15]. To address this, current advanced methods are utilizing self-training and weak supervision techniques to enhance data scaling by using model predictions as ground truth [11], [16]. However, a key drawback of these methods lies in their reliance on statistical confidence scores, which can lead to the generation of biologically implausible predictions (e.g., a *P. Vivax* species with Ring stage), as seen in the case of a teacher model predicting impossible configurations without adequate contextual validation which the student model ingest and reinforce [13], [22].

Consequently, current automated annotation methods often lack biological validity optimizing for pixel-wise coherence without ensuring that the resulting segmentation aligns with established biomedical taxonomy. Our proposed approach differentiates itself by integrating a formal semantic constraint mechanism via the NCBITaxon ontology [20]. By formally encoding domain rules (e.g., verifying $\exists$hasStage.*Trophozoite* is consistent with the predicted species), we ensure that machine-generated annotations are not just statistically probable, but biologically possible. This aligns with emerging hybrids of symbolic knowledge and deep learning [30], [31], offering a distinct advantage over purely heuristic or black-box annotation pipelines by guaranteeing that scalability does not come at the cost of clinical semantic fidelity.

## II. Materials and Methods

This study adopts a controlled, comparative experimental design to assess the influence of annotation source on the performance and interpretability of convolutional neural networks (CNNs) for malaria parasite classification. The methodology is structured around four interdependent components: (1) dataset annotation, (2) AI model training and evaluation, (3) semantic segmentation with ontological explanation, and (4) evaluation of performance and interpretability.

### A. Experimental Design

**Fig. 1.**
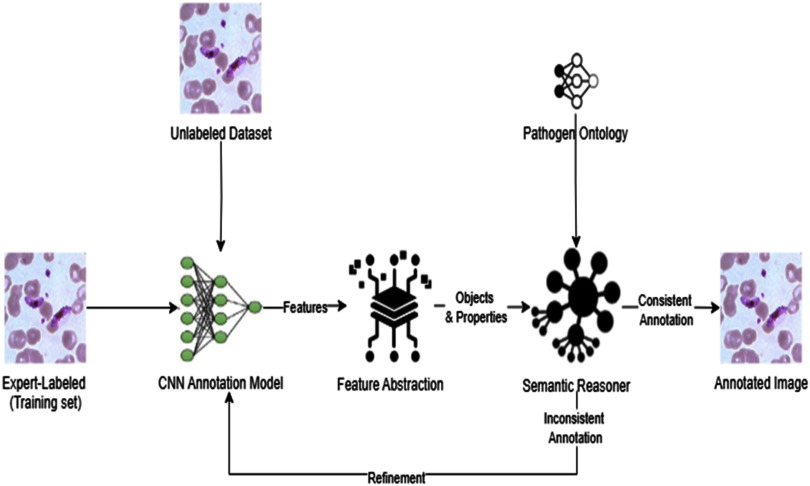
SegNet ontology annotation workflow process.

The central objective of this work is to evaluate whether machine-generated annotations derived through AI-assisted models with ontology-based reasoning and feedback can offer comparable diagnostic fidelity and semantic transparency to expert-generated annotations. To this end, three distinct annotation strategies are employed: (i) manual annotation by domain experts, (ii) SegNet Only annotation, and (iii) SegNet incorporating ontological reasoning and feedback. Each strategy was used to annotate the same underlying dataset to ensure comparability across models trained under consistent settings this is shown in Fig. 1. These annotation modalities form the basis for training identical CNN architectures, enabling a systematic comparison of model outputs under matched experimental conditions.

### B. Dataset and Annotation Strategies

The study leverages the Malaria Parasite Image Database (MP-IDB) as the foundational image source [17]. While the public MP-IDB repository contains 27558 high-resolution images annotated with bounding boxes for *Plasmodium* species and life-stage features. For this study, a balanced subset of 6000 images was used, distributed across four *Plasmodium* species (*P. falciparum, P. vivax, P. ovale, and P. malariae*) and three life stages (trophozoite, schizont, gametocyte). The selection process was stratified to avoid class imbalance. Images were resized to 256256 pixels, normalized, and augmented via rotation, contrast shifting, and zooming to improve model generalization. The choices align with the study's dual goals of achieving high segmentation performance and producing outputs that are interpretable and biologically coherent. The three annotation strategies were carried out as follows:

### C. Human Annotations

To establish a highly granular ground truth, three expert annotators who are trained pathologists with extensive experience in malaria microscopy manually re-annotated a curated 500-image seed subset drawn from the MP-IDB. Moving beyond the dataset's original bounding boxes, this custom annotation protocol involved the precise pixel-level masking of the *Plasmodium* parasites. Crucially, the experts delineated species and life-stage boundaries to provide the necessary training data for the CNN. Annotation was performed using Qupath [18]. Each image was reviewed by at least one expert, with ambiguous cases resolved through consensus discussion. Formal inter-annotator agreement statistics were not computed, given the exploratory and supportive role of these annotations. The focus was on presence and approximate localization rather than precise delineation, requiring approximately 35 minutes per image. These expert-derived annotations were treated as the ground truth and served as the gold standard for training, validating, and benchmarking the proposed method.

### D. SegNet Only Annotations

In the SegNet-only annotation strategy, a convolutional neural network (CNN) model based on the SegNet architecture was trained exclusively on a small subset of 500 expert-annotated microscopy images. The 500-image seed set was created via stratified sampling to maintain class balance across *P. falciparum, P. vivax, P. ovale,* and *P. malariae*, preventing class collapse in the initial training phase. This baseline model was then deployed to generate annotations across the remaining unannotated dataset without any additional semantic filtering or ontological validation. The segmentation outputs produced by SegNet classified parasite regions at the pixel level into *Plasmodium* species and intraerythrocytic life stages based solely on learned visual features. Unlike expert annotations or ontology-guided supervision, this approach operates in a fully data-driven manner, relying on the model's internal representations of morphology, chromatin distribution, and spatial structure to delineate cellular regions [19]. The annotations generated under this strategy reflect the model's raw capacity to infer biological entities from visual cues, thereby serving as a practical proxy for weak supervision pipelines commonly used in scalable diagnostic AI workflows. SegNet was selected for its proven efficacy in biomedical image segmentation tasks, particularly where fine spatial detail is critical, as in parasite morphology and as a baseline architecture to ensure that performance differences were attributable solely to the annotation regimes (Human vs. Machine) rather than architectural complexity. Its encoder-decoder architecture preserves spatial information through pooling index transfer, enabling precise boundary delineation. Using an identical architecture across annotation regimes isolates the effect of annotation quality on model performance, ensuring methodological fairness in comparative analysis.

### E. Proposed Semantic-Augmented (SegNet Semantic Reasoning) Annotation

The proposed annotation strategy used the same configuration and data as in the SegNet Only strategy, then Pellet semantic reasoner was added to filter out biologically implausible post-hoc. To ensure ontological rigor and biological plausibility, the machine-generated annotations were validated using formal reasoning over the NCBITaxon ontology, a comprehensive taxonomy of organisms maintained by the NCBI and widely adopted within the OBO Foundry for semantic data modeling in the life sciences [20]. While the NCBITaxon [20] provides the foundational taxonomic hierarchy for *Plasmodium* species, we custom-extended this ontology for the purpose of this study by encoding specific phenotypic axioms. This extended structured vocabulary allows the system to link the CNN's predicted diagnostic classes to their expected biological features (e.g., verifying that a CNN's visual detection of Maurer's clefts is biologically consistent with a concurrent prediction of *P. falciparum*). Each segmented parasite region was mapped to taxonomic concepts (e.g., NCBITaxon:5833 for *Plasmodium falciparum*) by representing model outputs as RDF triples. Logical assertions, such as $\exists$hasStage.Trophozoite and $\exists$hasSpecies.*P. falciparum*, were checked for coherence using Pellet reasoner. Any predictions found to violate taxonomic or stage-species constraints were filtered out. This process ensured the semantic validity of the annotations, facilitating the generation of structured, ontology-grounded training data. This process is shown in Fig. 1

### F. CNN Model Architecture and Training

Each annotation stream feeds into the training of a deep convolutional encoder-decoder network optimized for pixel-wise classification tasks in biomedical imaging [21]. The task of the model is to detect *Plasmodium* presence and perform a multi-class classification encompassing species (e.g., *P. falciparum, P. vivax*) and life stages (e.g., ring, schizont). The model relies on the macro-morphological pixel distributions of the bounding cells rather than the explicit sub-pixel localization of individual organelles. Each model was trained separately using the same hyper-parameters: Optimizer: Adam, Learning-rate: 1e-4, Batch size: 32, Epochs: 100, Loss function: categorical cross-entropy and augmentation-routines: shear-and-shift. Furthermore, 80:10:10 data split regiment was used. Training was conducted under the same controlled conditions for all three annotation streams to isolate the effect of annotation quality.

### G. Semantic Segmentation and Ontological Explanation

Because the CNN outputs pixel-level segmentation exclusively for Species and Life Stages, the reasoning engine acts as a semantic enrichment and verification layer. For instance, when the CNN classifies a segmented region as *P. falciparum* at the schizont stage, the Pellet reasoner validates this prediction against the ontology. It then generates a human-interpretable explanation by inferring the expected axiomatic properties of that class. By outputting assertions such as $\exists$hasStage.Schizont $\exists$hasExpectedFeature.Maurer'sCleft, the system provides the clinician with the formal biological justification underlying the prediction, without falsely implying that the CNN visually localized the sub-cellular organelle.

**TABLE I:** Summary of Results

METRIC	EXPERT	SEGNET ONTOLOGY	SEGNET-ONLY
DICE COEFFICIENT	0.86	0.83	0.71
INTERSECTION OVER UNION (IOU)	0.81	0.78	0.64
PIXEL-WISE ACCURACY	92.7	90.5	82.1
SEMANTIC GROUNDING ACCURACY	94.2	92.6	78.4
CLINICIAN TRUST SCORE (15)	4.7	4.4	3.6
FLEISS' KAPPA (INTERPRETABILITY)	0.81	0.77	0.61

Post-segmentation, model outputs were semantically grounded using a reasoning pipeline built on OWLRDF representations of the NCBITaxon ontology. SPARQL queries and the Pellet reasoner were used to infer species-stage relationships and to check for ontological consistency at this stage of the pipeline. Outputs were mapped to concepts such as $\exists$hasFeature.Maurer'sCleft or $\exists$hasStage.Trophozoite, providing clinically relevant justifications.

**Fig. 2.**
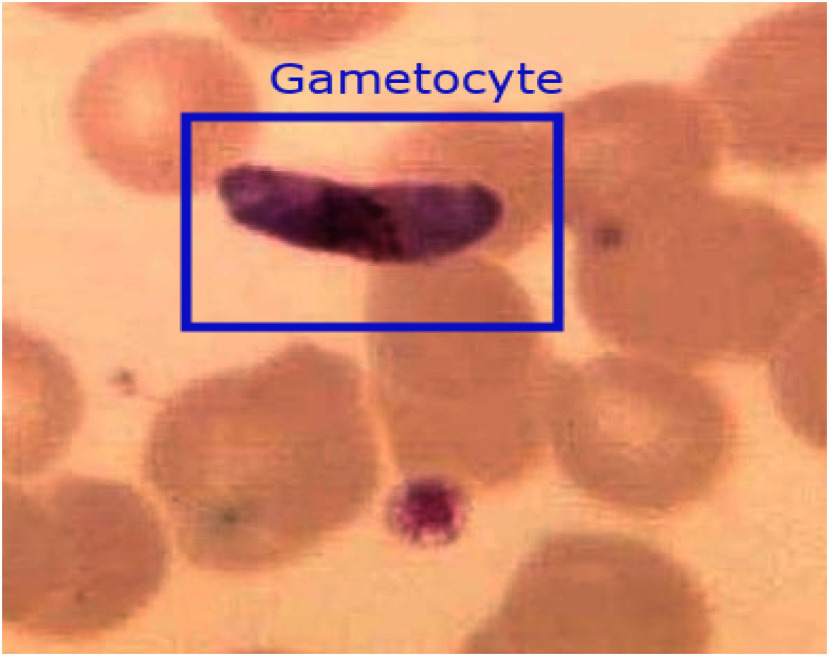
Example of a blood sample with gametocyte and nucleus. The semantic explanation generated for this sample is $\exists$has stage.gametocyte $\exists$has expected feature nucleus.

Each segmented object triggers logical assertions such as $\exists$hasFeature.Gametocyte or $\exists$hasStage.Trophozoite. These assertions are then processed by Pellet reasoning engine, which evaluates semantic consistency, performs class inferences, and generates human-interpretable explanations. For instance, when the CNN successfully segments a macro-region and classifies the bounding cell as a Gametocyte, the Pellet reasoner validates this prediction against the ontology. If the prediction does not violate the biological constraints of the inferred stage, the annotation is verified and enriched with its defining properties (e.g., $\exists$hasStage.Gametocyte $\exists$hasExpectedFeature.Nucleus), as shown in Fig. 2.

### H. Evaluation Strategy and Metrics

Model performance was assessed on a reserved 10 validation set using Pixel-wise Accuracy, Intersection over Union (IoU), Dice Coefficient, Mean Class Accuracy, and F1-score. Explanation fidelity was evaluated through semantic alignment rate, defined as the proportion of predictions consistent with biologically valid inference chains from the NCBITaxon Ontology.

A blinded review by 3 clinical parasitologists assessed explanation clarity, biological plausibility, and trustworthiness on a 5-point Likert scale. Inter-rater reliability (Fleiss ) quantified agreement, while trust scores captured user confidence. These qualitative assessments complemented quantitative metrics, providing an integrated evaluation of both technical performance and real-world applicability.

## III. Results

**Fig. 3.**
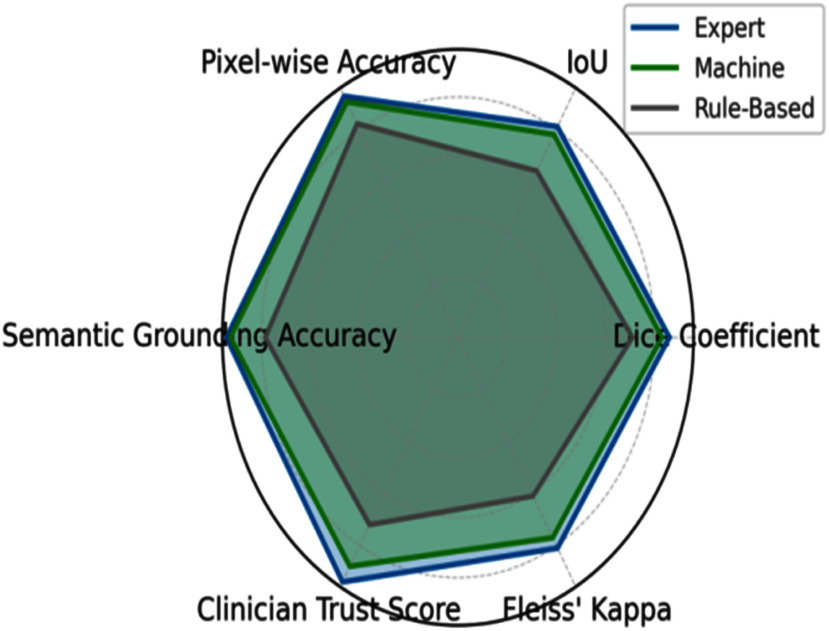
Radar chart for the performance of the different annotation strategies.

The results presented in this section provide a comparative evaluation of the performance and interpretability of the convolutional neural network (CNN) model trained on three distinct annotation strategies: (i) manual expert annotation, (ii) SegNet-only annotation, and (iii) Proposed SegNet Semantic-augmented annotations refined through ontological feedback here after referred to as SegNetOntology. These three metrics are presented in Fig. 3 as Expert, Rule based and Machine respectively.

### A. Segmentation Accuracy

The comparative performance of the models revealed important trade-offs in segmentation accuracy. The expert-annotation achieved the highest overall accuracy, with a mean IoU of 0.81, Dice coefficient of 0.86, and pixel-wise accuracy of 92.7 across all *Plasmodium* species and life stages. The SegNetOntology model demonstrated highly competitive results, with IoU of 0.78, Dice of 0.83, and accuracy of 90.5. In contrast, the SegNet-only model showed significantly lower performance (IoU 0.64, Dice 0.71, Accuracy 82.1). Statistical analysis using ANOVA with Tukey's HSD test confirmed that the difference between the expert and SegNetontology models did not reach statistical significance (p0.09) in this sample, suggesting the SegNetontology model offers a viable, lower-resource alternative without evidence of a statistically significant performance degradation in this sample, whereas both significantly outperformed the SegNet-only model (p < 0.01).

### B. Semantic Grounding Accuracy

Semantic coherence was assessed by validating model outputs against the NCBITaxon ontology using SPARQL queries and Pellet-based OWL reasoning. The expert-annotation achieved the highest semantic fidelity, with 94.2 of predictions aligning with valid ontological constraints. The SegNetOntology model followed closely at 92.6, while the SegNet Only model lagged at 78.4. Notably, semantic post-processing identified and filtered out 5.7 of AI-generated inconsistencies. By discarding predictions that contained conflicting Stage-Species assertions, the system maintained ground-truth purity, illustrating the utility of integrating structured knowledge to regulate weakly supervised pipelines. The performance of the SegNetontology model indicates that semantic refinement effectively compensates for noise (predictions violating NCBITaxon constraints) introduced during initial annotation, producing outputs with biological plausibility comparable to human-generated annotations. Noise here were instances where the model prediction conflicted with the ontology (e.g., conflicting Species-Stage assertions), and the reasoner filtered them, thereby quantifying the noise reduction.

### C. Human-Centered Explainability

To provide a preliminary qualitative assessment of explainability, three (3) clinical parasitologists reviewed segmented images from all three models in a blinded study. Explanations were rated based on three criteria: 1. Clarity of the predicted life-stage and species, 2. Trust in the classification process, 3. Perceived diagnostic utility of the explanations.

Expert-annotation received an average trust score of 4.75, with 87 agreement (Fleiss' 0.81). The SegNetontology model achieved a trust score of 4.45 and 82 agreement ( 0.77), showing high clinician confidence despite its automated origin. The SegNet-only model was perceived as less interpretable, scoring 3.65 with 68 agreement ( 0.61). Reviewers noted that the semantic assertions generated by the proposed pipeline (e.g., $\exists$hasStage.Gametocyte $\exists$hasSpecies.*P.falciparum*) added transparency and justification, especially in the machine-annotated setting.

These results collectively demonstrate that Semantic-augmented annotations when reinforced by semantic reasoning can approximate human-level diagnostic performance while reducing the burden of manual annotation. The burden is alleviated by requiring experts to annotate only a small "seed" dataset (500 images) to train the initial teacher model, rather than the full dataset (6000 images). The ontology then acts as an automated "supervisor" for the remaining data, reducing the need for human intervention. Moreover, the use of domain ontologies ensures that even automatically generated outputs retain interpretive clarity, enabling a trustworthy AI pipeline for malaria diagnosis. The findings contribute to a growing body of evidence that semantic supervision and ontological integration are critical enablers of scalable, explainable medical AI systems [22], [23], [24].

## IV. Discussion

The findings in this work demonstrate that, when coupled with ontological feedback, machine-generated annotations can approach the accuracy and interpretability of expert-annotated data, thereby offering a scalable and viable alternative for resource-limited settings. However, the marginal differences observed between the expert-annotation and the proposed models, especially in Dice coefficient (0.86 vs. 0.83) and IoU (0.81 vs. 0.78) suggest that annotations augmented with ontological validation retain significant diagnostic utility in cost and effort. This supports recent claims that weakly supervised learning, when properly constrained semantically, can rival fully supervised approaches [15], [25]. Thus, the ontology ensures that these granular visual detections are harmonized with established taxonomic constraints, the framework guarantees that clinicians receive explanations bounded by established biological reality, moving beyond black-box opacity without requiring exhaustive sub-pixel annotations.

Crucially, semantic grounding accuracy remained high for both expert and SegNetontology generated annotations (>92), showing that ontology-based constraints can serve as a form of regularization that enforces biological plausibility. This is particularly relevant in malaria diagnosis, where subtle morphological differences, such as between *P. falciparum* trophozoites and *P. vivax* schizonts can influence treatment decisions. By integrating NCBITaxon-based reasoning into the feedback loop, the model avoids generating ontologically inconsistent or clinically irrelevant predictions, echoing findings from similar work in pathology [13], [26].

Perhaps most notably, the clinician-centered evaluation of explanation quality revealed that the SegNetontology model achieved high trust scores (4.45) and interpretability ratings ( 0.77), rivaling the expert-annotation pipeline. This underscores the potential of structured explanations formulated as OWL-class assertions like $\exists$hasStage.Gametocyte and $\exists$hasSpecies.*P.vivax* to bridge the gap between black-box AI and clinically meaningful outputs. Such human-aligned semantic scaffolding appears to mitigate common concerns around opacity and trustworthiness in medical AI [14], [22].

In contrast, the SegNet Only (baseline) annotation model underperformed across all metrics, highlighting the limitations of rigid, mathematical heuristics in complex biological domains. These results suggest that machine supervision, when grounded in a well-structured ontology and refined through semantic reasoning scales well in cases with high intra-class variability and class imbalance, which are common in field-collected malaria samples.

Overall, this study contributes to the emerging discourse on explainable medical AI by demonstrating a functional hybrid approach: combining CNN-based pixel learning with ontology-driven post-hoc explanation. It builds on prior work that has called for interpretable, domain-aware models [13], [27], while presenting a novel application in malaria diagnosis a domain where timely, scalable annotation remains a persistent challenge.

Importantly, we formalize this approach as *Knowledge-Augmented Explainability*. These explanations do not imply mechanistic, pixel-level visual detection of sub-cellular biological structures (which is often impossible given the resolution of standard thin-smear datasets). Instead, the system leverages the CNN's statistical prediction of the macro-cell to anchor a formal deductive proof. The ontology guarantees that clinicians receive explanations enriched by established biological taxonomy, moving beyond black-box opacity without fabricating granular visual evidence.

This reduction in annotation effort is consistent with emerging paradigms in broader biomedical image analysis, where "human-in-the-loop" bottlenecks are increasingly being replaced by knowledge-driven automation. For instance, in the domain of whole-cell segmentation, [28] demonstrated that deep learning models could achieve human-level performance across diverse tissue types by leveraging large-scale data with computationally efficient annotation strategies, rather than relying solely on manual pixel delineation. Similarly, [29] showed that low-quality annotations could be algorithmically upgraded to reduce manual costs while maintaining segmentation fidelity. Our findings extend this logic to malaria diagnostics, suggesting that ontological constraints can serve as the "upgrade" mechanism for noisy annotations.

Furthermore, parallels exist in radiology, where [12]. utilized pre-trained models to automate data annotation for large datasets, significantly accelerating pipeline development. However, unlike general radiology tasks where visual features often suffice, malaria diagnosis requires strict adherence to biological taxonomies (e.g., specific stage-species combinations). By enforcing these constraints via the NCBITaxon ontology, our usage model effectively filters the "noise" inherent in automated annotation which is a challenge also noted in recent surveys on data synthesis. This confirms that integrating domain-specific structured knowledge is a viable pathway to replicate the annotation-efficiency gains seen in cell biology and radiology within the more semantically constrained environment of infectious disease diagnosis.

## V. Limitations

While the proposed ontology-refined annotation framework demonstrates strong performance and scalability, several limitations warrant consideration. First, the evaluation relied on the MP-IDB dataset, which, although has high quality, may not capture the full spectrum of imaging variability encountered in field diagnostics, such as differences in staining quality, microscope hardware, and operator technique. Second, the ontology reasoning process was limited to the NCBITaxon hierarchy; broader integration with complementary ontologies (e.g., IDOMAL for disease stages, SYMP for symptom mapping) could further enhance semantic constraints. Third, the comparison focused exclusively on SegNet, leaving open the question of whether other modern segmentation architectures, such as U-Net variants or transformer-based models, might benefit even more from ontology integration. Finally, clinician trust and interpretability assessments were based on a limited pool of expert reviewers (three), which may not fully represent the diversity of perspectives. These factors suggest avenues for further validation and refinement.

## VI. Implications

The findings in this work has significant implications for scaling AI-assisted malaria diagnostics in resource-limited, high-burden regions where expert annotation capacity is scarce. By integrating formal biomedical ontologies into the annotation pipeline, the approach ensures that outputs are not only statistically accurate but also biologically coherent, thereby increasing clinical interpretability and trust [30]. The methodology offers a replicable framework for other vector-borne diseases such as Dengue, and Zika, where similar annotation and interpretability challenges persist. Furthermore, the demonstrated compatibility of ontology reasoning with deep learning architectures like SegNet suggests a pathway toward hybrid AI systems that unify domain knowledge and data-driven learning. Such systems hold promise for improving the reliability, transparency, and equity of automated diagnostic tools in global health.

## VII. Conclusion

This study provides an investigation into the comparative utility of human and SegNetontology generated annotations for training convolutional neural networks in the semantic segmentation of *Plasmodium* species and life-cycle stages from thin smear blood images. By integrating ontological reasoning through structured biomedical vocabularies such as NCBITaxon, the research highlights the potential for semantic technologies to enhance model interpretability, diagnostic consistency, and trustworthiness which are key factors in clinical deployment.

Our findings reaffirm the enduring value of expert annotations as a gold standard, especially in achieving pixel-level precision and ontologically grounded classification [31]. However, the results also demonstrate that machine-generated annotations when guided by structured feedback and semantic constraints can approximate expert performance in both accuracy and clinical explainability. Furthermore, the study contributes to the growing body of work on hybrid AI systems that fuse deep learning with symbolic knowledge representation.

Future research could build on these findings by incorporating active learning frameworks, implementing cross-ontology mappings with IDO and SNOMED CT, and extending the methodology to other infectious diseases such as Dengue, and Zika. Such advancements have the potential to significantly alleviate the annotation bottleneck while preserving trust and transparency in AI-driven diagnostics. Additional directions include dynamic annotation refinement, real-time ontological inference, and evaluating the transferability of these frameworks to broader pathological, parasitological and histopathological applications.

## Code Availability

The code for this work and additional resources can be found at: https://colab.research.google.com/drive/1N3TWygb0DGZRU5laosIY1kzPzhIPQTCk

## Conflict Interest Disclosure

The authors have no conflict of interest to disclose.
